# ﻿Two new records and an updated checklist of freshwater crabs (Arthropoda, Malacostraca, Decapoda, Potamidae and Gecarcinucidae) from Bangladesh

**DOI:** 10.3897/zookeys.1167.102766

**Published:** 2023-06-16

**Authors:** Shibly Sadique Shashi, Da Pan, Nusrath Jahan Emu, Mishal Roy, Md. Abu Sadek, S M Sharifuzzaman, Hongying Sun

**Affiliations:** 1 College of Life Sciences, Nanjing Normal University, Nanjing 210023, China; 2 Institute of Marine Sciences, University of Chittagong, Chattogram 4331, Bangladesh; 3 Department of Fisheries, University of Chittagong, Chattogram 4331, Bangladesh

**Keywords:** *Acanthopotamon*, biodiversity, conservation, *Maydelliathelphusa*, south Asia

## Abstract

The species diversity of freshwater crabs of Bangladesh is poorly known. In this study, *Acanthopotamonfungosum* (Alcock, 1909) and *Maydelliathelphusaedentula* (Alcock, 1909) are reported as new records from Bangladesh. The two species were identified through morphology and molecular phylogeny based on 16S rDNA gene sequences. Herein, diagnostic characters of both species are provided respective to close congeners. There is concern over the conservation status of *A.fungosum* due to its narrow distributional range. In addition, an updated checklist and a key to the freshwater crabs of Bangladesh are provided.

## ﻿Introduction

Freshwater crabs are common in the tropical and subtropical regions where they inhabit a wide range of habitats such as rivers, swamps, lakes, and caves (Yeo et al. 2008). Bangladesh is a riverine country and has various bodies of freshwater suitable for crabs. For example, the hilly areas of the districts of Khagrachari, Rangamati, Bandarban, Chattogram, Cox’s Bazar, Mymensingh, Netrokona, Sylhet, Moulvibazar, and Habiganj have watersheds consisting of rivers, lakes, streams, and waterfalls that harbour unique aquatic biodiversity, including crabs. The wetlands at Manikganj, Jessore, Narail and Rajshahi, and the Halda and Meghna rivers are also suitable habitats for crabs (Hasan and Rashid 2016).

More than 1,300 species of freshwater crabs have been found throughout the world (Yeo et al. 2008). Remarkably, only five species, namely *Acanthopotamonmartensi* (Wood-Mason, 1875) [recorded as *Potamonmartensi*], *Lobothelphusawoodmasoni* (Rathbun, 1905) [recorded as *Potamonwoodmasoni*], *Lamellalamellifrons* (Alcock, 1909) [recorded as *Paratelphusalamellifrons*], *Sartorianaspinigera* (Wood-Mason, 1871) [recorded as *Paratelphusaspinigera*], and *Sartorianatrilobata* (Alcock, 1909), have been reported from Bangladesh (Shafi and Quddus 1982; Siddiqui and Zafar 2002; Ahmed et al. 2008; Rahman et al. 2008; Hossain 2015; Mia et al. 2016; Hasan and Rashid 2016; Chakraborty et al. 2021). There are two additional reports, i.e., *Austrothelphusatransversa* (von Martens, 1868) (Ahmed et al. 2008; Rahman et al. 2008; Hossain 2015; Chakraborty et al. 2021) and *Pyxidognathusfluviatilis* (Alcock, 1900) (Ahmed et al. 2008; Rahman et al. 2008; Hossain 2015; Hasan and Rashid 2016; Chakraborty et al. 2021), but the former is native to Australia and Papua New Guinea (Esser and Cumberlidge 2008) and the latter species inhabits fresh, brackish, and marine waters and is not regarded as true freshwater crab (Yeo et al. 2008). Therefore, the record of *A.transversa* is inaccurate or questionable. Overall, the species diversity of freshwater crabs of Bangladesh and their natural history remains largely unknown. Based on morphological and molecular data, the present study newly reports *Acanthopotamonfungosum* (Alcock, 1909) and *Maydelliathelphusaedentula* (Alcock, 1909) as part of freshwater crab fauna of Bangladesh.

## ﻿Material and method

**Figure 1.**
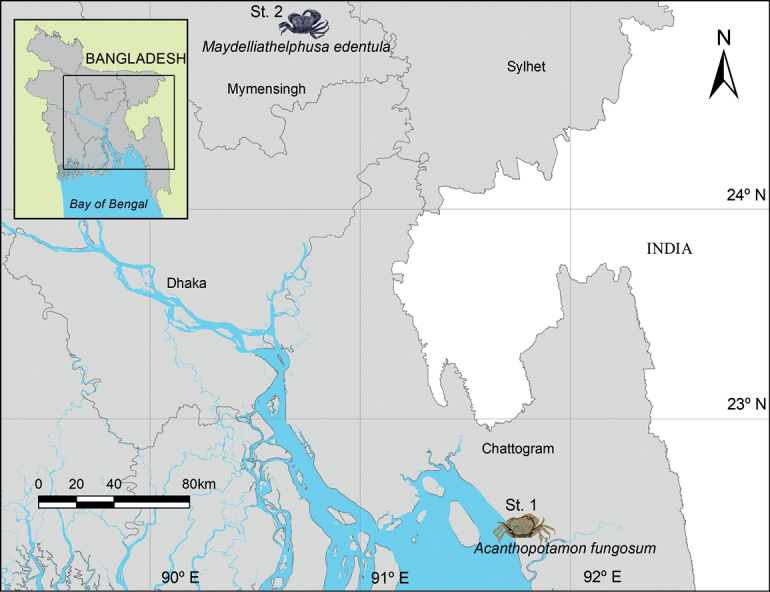
Map showing the collection points of two crab species from Bangladesh. Station 1: *Acanthopotamonfungosum* collected from Chittagong University Campus, Chattogram. Station 2: *Maydelliathelphusaedentula* collected from the Kangsa River, Mymensingh.

During a field survey in August 2021, two specimens of freshwater crab were collected from a small hilly stream near the Chittagong University Campus, Chattogram and from the Kangsa River, Mymensingh (Fig. 1). Crabs were collected by hand and with a fishing net. Both specimens were photographed, diagnosed based on their morphometric characters, preserved in 95% ethanol, and had tissue sampled for molecular study. These specimens were transferred to the laboratory of Institute of Marine Sciences (IMS), University of Chittagong (CU) for further study. The specimens were identified following the taxonomic keys of Pati et al. (2019) and Das (2021). The two crab specimens were deposited in the IMSCU (voucher numbers IMSCU/FW-crab2108.01 and IMSCU/FW-crab2108.02) for future reference. The following abbreviations used are: G1 for the male first gonopods; G2 for the male second gonopods. The terminology of morphological characteristics used follows that of Ng (1988) and Davie et al. (2015).

Genomic DNA was extracted from the gill tissue using Trelief Animal Genomic DNA kit (Tsingke, China) according to the manufacturer’s protocol and sequenced with an Illumina HiSeq X Ten platform (150 bp paired end). Full-length mitochondrial 16S sequences were assembled using MITOZ (Meng et al. 2019). Sequences were uploaded to NCBI GenBank with accession numbers OQ788486 and OQ788487. Together with downloaded sequences, the matrix was aligned with MAFFT (Katoh and Standley 2013) using the default setting. IQ-TREE (Nguyen et al. 2015) was employed to conduct a maximum-likelihood (ML) analysis. Node supports were obtained through 1,000 ultra-fast bootstrap replicates (Minh et al. 2013). The phylogenetic tree was visualized using ITOL (Letunic and Bork 2019).

## ﻿Taxonomy

### ﻿Family Potamidae Ortmann, 1896

#### ﻿Genus Acanthopotamon

Taxon classificationAnimaliaDecapodaPotamidae

Kemp, 1918

C88662EB-93A3-5E2A-BA94-A4B8808BB3CC

##### Type species.

*Paratelphusamartensi* Wood-Mason, 1875, by original designation.

#### ﻿ Acanthopotamon fungosum

Taxon classificationAnimaliaDecapodaPotamidae

(Alcock, 1909)

B66E39EE-A8CC-56BC-93FE-115CCF0E32CD

Figs 2 , 4a, b

Potamon (Paratelphusa) fungosum Alcock, 1909: 250.
Potamon (Acanthopotamon) fungosum —Alcock 1910: 65, fig. 12.
Lobothelphusa fungosa —Bott 1970: 148, pl. 38 fig. 25, pl. 46 fig. 23.
Paratelphusa fungosum —Brandis and Sharma 2005: 14.
Acanthopotamon fungosum —Yeo and Ng 2007: 274; Ng et al. 2008: 159.

##### Material examined.

1 male, 28.84 × 24.28 mm (Table 1), Chittagong University Campus, Chattogram, Bangladesh, 22°28'07"N, 91°46'48"E; 15 August 2021, collected by Shibly Sadique Shashi.

**Table 1.** The morphometric features of *Acanthopotamonfungosum* (IMSCU/FW-crab2108.01) and *Maydelliathelphusaedentula* (IMSCU/FW-crab2108.02). Lengths and widths in mm; weight in g.

Morphometric data	*Acanthopotamonfungosum* (♂)	*Maydelliathelphusaedentula* (♂)
Carapace width	28.84	65.89
Carapace length	24.28	49.16
Frontal width	9.11	14.84
Pleon width	11.13	24.72
Pleon length	15.89	35.29
Telson length	4.26	10.37
Major chela length	22.23	65.52
Cheliped length	41.65	125.57
Dactyl length	13.81	45.77
Merus length	10.44	25.97
1^st^ ambulatory leg length	33.31	72.96
2^nd^ ambulatory leg length	38.99	88.87
3^rd^ ambulatory leg length	38.01	84.55
4^th^ ambulatory leg length	32.24	65.64
Weight	5.99	97.00
No. of epibranchial tooth on each side	4	1

##### Description of the male.

**Figure 2.**
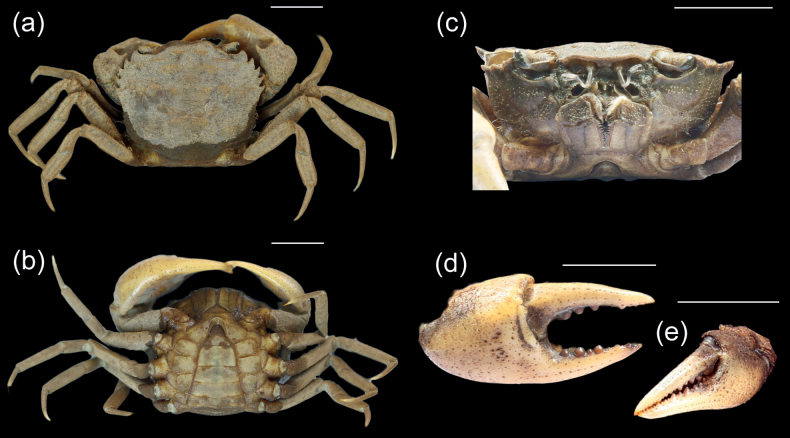
*Acanthopotamonfungosum***a** dorsal view **b** ventral view **c** frontal view **d** major chela **e** minor chela. Scale bars: 1 cm.

Carapace subhexagonal, convex, covered by short spongy fur, dorsal surface rough, region distinct; ca 1.19× broader than long. H-shaped groove distinct. Epigastric cristae broad, blunt, well advance of postorbital cristae; post orbital cristae short, not confluent with first epibranchial tooth (Fig. 2a); external orbital tooth blunt, broadly triangular; anterolateral margin convex, with 4 epibranchial teeth, first epibranchial tooth distinctly triangular, others sharp. Eyes moderate in size, outer margin of eye with U- to V-shaped incision (Fig. 2c). Third maxilliped elongated, rectangular; with well-developed flagellum present distally on exopod; exopod not distally tapering and longer than merus width. Epistome lateral margins slightly sinuous, medial lobe triangular (Fig. 2c).

Chelipeds unequal in size, right larger; carpus and merus of cheliped with distinct subdistal and subterminal spine, fingers longer than palm, distinct gap with dactyl and pollex closed, both movable and immovable fingers with 3 or 4 large, rounded teeth (Fig. 2d); fingers of minor chela slightly gaping when closed (Fig. 2e). Ambulatory legs bearing short setae; second pair of ambulatory legs longest, fourth pair shortest; dactylus slender, styliform, with spinules (Fig. 2a).

Thoracic sternum smooth, pitted, suture between s1/s2 completely fused to form triangular structure; suture between s3/s4 indistinct, suture between s4/s5, s5/s6, s6/s7, s7/s8 distinct (Fig. 2b).

Pleon broadly triangular; all segments rectangular. Telson tongue-shaped, length and width almost equal (Fig. 2b).

G1 curved outwardly, gradually tapering towards tip; terminal segment subcylindrical, slender, covered by short setae; nearly 3× shorter than subterminal segment (Fig. 4a, b). G2 elongated, shorter than G1.

##### Remarks.

*Acanthopotamonfungosum* was originally described as Potamon (Paratelphusa) fungosum Alcock, 1909 from Cachar, India (Bott 1970). Previously, *A.fungosum* was only known from the states of Arunachal Pradesh, Assam, Mizoram and Manipur, in India (Pati et al. 2019; Mitra and Pati 2021; Rath et al. 2022). This species is recorded here for the first time from Bangladesh. *Acanthopotamonmartensi* is also distributed in Bangladesh, i.e., in Manikganj district and estuaries of the Chakaria Sundarban area (Shafi and Quddus 1982; Rahman et al. 2008).

The IUCN conservation status of *A.fungosum* was assessed as Data Deficient (DD) (Cumberlidge 2008a). The species is distributed over a small geographical area, i.e., eastern Bangladesh and southern Assam, India. Due to restricted distributional range and increasing threats to freshwater habitats of this region from various human activities, *A.fungosum* is likely more threatened than *M.edentula*. Further field surveys are needed to determine population size and threats.

Until now, four species have been described for the genus *Acanthopotamon* (Pati et al. 2019). *Acanthopotamonfungosum* can be easily differentiated by having four epibranchial teeth, compared to two epibranchial teeth in *A.panningi* (Bott 1970: fig. 19; Pati et al. 2019), three in *A.horai* (Pati et al. 2019), and three in *A.martensi* (Bott 1970: fig. 20; Rahman et al. 2008; Pati et al. 2019).

### ﻿Family Gecarcinucidae Rathbun, 1904

#### ﻿Genus Maydelliathelphusa

Taxon classificationAnimaliaDecapodaGecarcinucidae

Bott, 1969

6E02AB29-5ECA-50A0-BE9E-4FAB4A5C6EAB

##### Type species.

*Telphusamasoniana* Henderson, 1893, by original designation.

#### ﻿ Maydelliathelphusa edentula

Taxon classificationAnimaliaDecapodaGecarcinucidae

Alcock, 1909

D78E2F36-7B28-544F-A5F9-AE3FBC8BC29B

Figs 3 , 4c, d

Potamon lugubre edentulum Alcock, 1909: 247.
Paratelphusa (Barytelphusa) edentula —Alcock 1909: 376; Alcock 1910: 84, fig. 19.
Barytelphusa (Maydelliathelphusa) edentula —Bott 1970: 34.
Maydelliathelphusa edentula —Ng et al. 2008: 68.

##### Material examined.

1 male, 65.89 × 49.16 mm (Table 1), Kangsa River, Netrokona, Mymensingh, Bangladesh, 25°00'45"N, 90°38'54"E, 10 August 2021, collected by Shibly Sadique Shashi and Nusrath Jahan Emu.

##### Description of the male.

**Figure 3.**
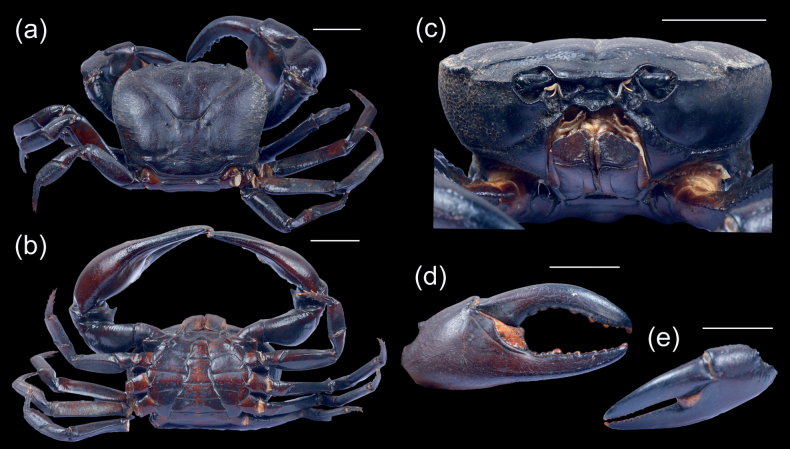
*Maydelliathelphusaedentula***a** dorsal view **b** ventral view **c** frontal view **d** major chela and **e** minor chela. Scale bars: 2 cm.

Carapace slightly depressed, ca 1.34× broader than long; epigastric cristae distinct, epigastric and postorbital cristae on either side united (Fig. 3a); postorbital cristae distinct, sharp, subparallel to frontal margin; frontal region deflexed, relatively wide; external orbital tooth prominent, epibranchial tooth present, prominent; frontal margin bilobed, frontal median triangle not complete; cervical groove well developed; mesogastric furrow very distinct, deep, slightly bifurcated posteriorly. Anterolateral and posterolateral regions rugose. Eyes smaller than orbital floor; eyestalk short. Third maxilliped with ischium subrectangular, longer than broad, with distinct narrow medial groove; merus pentagonal, broader than long; exopod slender, longer than ischium, reaching base of merus, with long flagellum (Fig. 3c). Epistome lateral margins slightly sinuous, medial lobe triangular (Fig. 3c).

Chelipeds surface smooth, unequal, right cheliped larger; carpus with distinct spine on inner angle; fingers longer than palm, movable finger strongly curved downward, immovable finger smoothly curved upward, wide gap between dactyl and pollex when closed, movable finger comparatively larger than immovable finger, inner margin of fingers lined with numerous round and blunt teeth (Fig. 3d); ambulatory legs stout; second pair of ambulatories longest while the fourth pair shortest (Fig. 3b); dactylus slender, longer than propodus, with 4 rows of spines on the margin (Fig. 3b).

Male thoracic sternum smooth, pitted. Sternites s1/s2 completely fused forming triangular structure; suture between s3/s4 shallow; suture between s4/s5, s5/s6, s6/s7, s7/s8 distinct (Fig. 3b).

Male pleon T-shaped, somites 5 and 6 constricted medially. Telson tongue-shaped, length and width almost equal (Fig. 3b).

**Figure 4.**
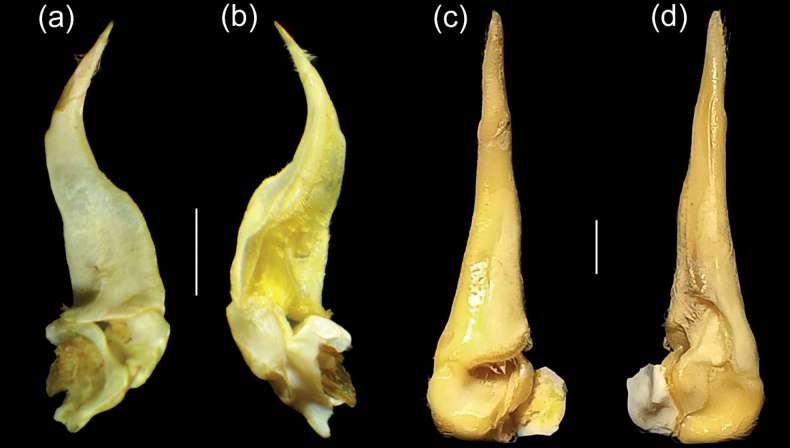
G1 **a, b***Acanthopotamonfungosum***c, d***Maydelliathelphusaedentula*. Scale bars: 2 mm.

G1 stout, straight; terminal segment tapering gradually, almost 2× shorter than subterminal segment (Fig. 4c, d). G2 elongated, shorter than G1.

##### Remarks.

*Maydelliathelphusaedentula* was originally described as Potamonlugubrevar.edentulumAlcock 1909 from Assam, India and subsequently transferred to Barytelphusa (Maydelliathelphusa) Bott, 1969 (Bott 1970). The present record is the first report of the genus *Maydelliathelphusa* from Bangladesh. Previously, *M.edentula* was documented in India (Assam, Nagaland, Mizoram) and Bhutan (Samchi) (Cumberlidge 2008b; Valarmathi 2017; Ray et al. 2018; Das 2021). Suitable habitats include freshwater rivers and streams (Cumberlidge 2008b). Therefore, it is not surprising that *M.edentula* occurs in eastern Bangladesh, near its known distribution range.

The IUCN conservation status of *M.edentula* is Near Threatened (NT) because of its limited distribution range and vulnerable habitat (Cumberlidge 2008b). Although the current study has expanded its known geographic distribution, the conservation status of this species is still not optimistic. In western Bangladesh, some local people, especially fishermen, eat this crab on a limited scale. In addition, various types of human activities, like pollution, urbanization and sand mining are impacting freshwater habitats in that area and, consequently, are threats to the population of *M.edentula*.

Until now, five species belonging to the genus *Maydelliathelphusa* have been recorded from India, Bhutan, and Nepal (Cumberlidge 2008c; Valarmathi 2017; Das 2021). The morphology of the carapace of *M.edentula* is superficially similar to that of other four species. However, *M.edentula* can be easily distinguished externally by its large, distinctively asymmetric chelipeds and its united epigastric and postorbital cristae (Bott 1970; Das 2021).

### ﻿Check list

**Figure 5.**
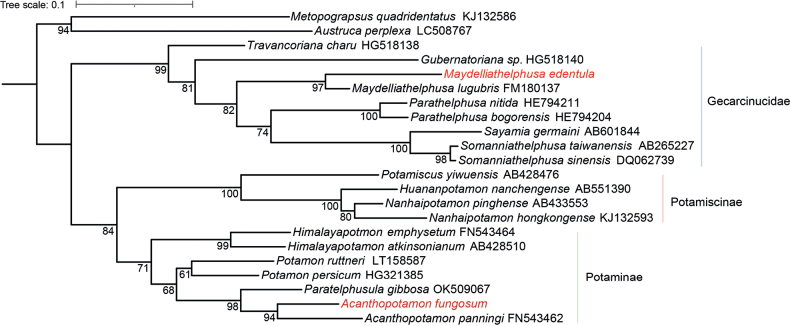
Reconstructed maximum-mikelihood tree based on 16S rDNA sequences. Newly obtained sequences are shown in red. Numbers on branches represent bootstrap values.

Herein, two freshwater crab species, *A.fungosum* and *M.edentula*, are documented for the first time from Bangladesh. A molecular phylogeny based on 16S rDNA sequences confirmed their identification (Fig. 5). Specifically, *A.fungosum* and *M.edentula* are clustered separately with *A.panningi* and *M.lugubris*. With these two species included, there are now seven species of true freshwater crab known to inhabit Bangladesh, namely *Acanthopotamonfungosum*, *A.martensi*, *Lobothelphusawoodmasoni*, *Lamellalamellifrons*, *Maydelliathelphusaedentula*, *Sartorianaspinigera*, and *S.trilobata* (Table 2).

**Table 2.** Checklist of the freshwater crabs from Bangladesh, with the inclusion of species identified in this study.

Family/genus/species	Locality	IUCN Status	References
Family Gecarcinucidae Rathbun, 1904			
Genus *Lamella* Bahir & Yeo, 2007			
1) *Lamellalamellifrons* (Alcock, 1909)^a^	5, 7, 8, 10, 11, 13	—	D–F
Genus *Maydelliathelphusa* Bott, 1969			
2) *Maydelliathelphusaedentula* (Alcock, 1909)*	5	—	This study
Genus *Sartoriana* Bott, 1969			
3) *Sartorianaspinigera* (Wood-Mason, 1871)^b^	1, 4, 7, 8, 9, 10, 12	Least Concern	A–C, E, G, H
4) *Sartorianatrilobata* (Alcock, 1909)	1, 2, 3	—	A
Family Potamidae Ortmann, 1896			
Genus *Acanthopotamon* Kemp, 1918			
5) *Acanthopotamonfungosum* (Alcock, 1909)*	6	—	This study
6) *Acanthopotamonmartensi* (Wood-Mason, 1875)^c^	4, 8, 11, 12	Least Concern	B–E, G, H
Genus *Lobothelphusa* Bouvier, 1917			
7) *Lobothelphusawoodmasoni* (Rathbun, 1905)^d^	1, 2, 3, 4, 11, 12	Least Concern	A–E, G, H

*Indicates a new record. Originally reported as: ^a^*Paratelphusalamellifrons* (see Shafi and Quddus 1982; Siddiqui and Zafar 2002; Mia et al. 2016); ^b^*Paratelphusaspinigera* (see Shafi and Quddus 1982); ^c^*Potamonmartensi* (see Shafi and Quddus 1982; Siddiqui and Zafar 2002); ^d^*Potamonwoodmasoni* (see Shafi and Quddus 1982; Siddiqui and Zafar 2002). Locality: 1: Cox’s Bazar; 2: Bandarban; 3: Maulovi Bazar; 4: Manikganj; 5: Mymensingh; 6: Chittagong University Campus, Chattogram; 7: Rajshahi; 8: Jessore; 9: Narail; 10: Sylhet; 11: Chakaria Sundarban; 12: Mogra River, Netrokona; 13: Dhaka. References: A: Hasan and Rashid (2016); B: Rahman et al. (2008); C: Hossain (2015); D: Siddiqui and Zafar (2002); E: Shafi and Quddus (1982); F: Mia et al. (2016); G: Ahmed et al. (2008); H: Chakraborty et al. (2021).

### ﻿Key to freshwater crabs from Bangladesh

**Table:** 

1	Mandibular palp with single terminal lobe; male pleon T-shaped	**2**
–	Mandibular palp with bilobed terminal segment; male pleon triangular	**5**
2	Epibranchial tooth situated well posterior to level of postorbital cristae, at widest part of carapace; G1 subterminal segment relatively broad	**3**
–	Epibranchial tooth situated in line with or slightly posterior to level of postorbital cristae, anterior to widest part of carapace; G1 subterminal segment relatively narrow	**4**
3	Anterolateral margin without protrusion between external orbital angle and epibranchial tooth; cervical groove relatively narrow; G1 terminal segment straight	***Sartorianaspinigera***
–	Anterolateral margin with one lobe-like protrusion between external orbital angle and epibranchial tooth; cervical groove relatively wide; G1 terminal segment bend outward	***Sartorianatrilobata***
4	Epigastric cristae sharp; outer margin of external orbital angle comparatively long; G1 with inner margin characteristically curved or angled just below juncture between terminal and subterminal segment	***Lamellalamellifrons***
–	Epigastric cristae rugose; outer margin of external orbital angle comparatively short; G1 straight	***Maydelliathelphusaedentula***
5	Carapace dorsal surface uneven, setose; male sternopleonal cavity reaches up to the level of the imaginary line joining the anterior part of the cheliped coxae	**6**
–	Carapace dorsal surface smooth, glabrous; male sternopleonal cavity reaches up to the level of the imaginary line joining the median part of the cheliped coxae	***Lobothelphusawoodmasoni***
6	Carapace with four epibranchial teeth on each anterolateral margin	***Acanthopotamonfungosum***
–	Carapace with three epibranchial teeth on each anterolateral margin	***Acanthopotamonmartensi***

References: Bahir and Yeo (2007); Yeo and Ng (2012); Pati et al. (2019); Chetia et al. (2021).

## Supplementary Material

XML Treatment for Acanthopotamon

XML Treatment for Acanthopotamon fungosum

XML Treatment for Maydelliathelphusa

XML Treatment for Maydelliathelphusa edentula

## ﻿Additional information

### Author ORCIDs

Da Pan https://orcid.org/0000-0001-5445-6423

Mishal Roy https://orcid.org/0000-0001-7560-4392

SM Sharifuzzaman https://orcid.org/0000-0003-1280-7947

Hongying Sun https://orcid.org/0000-0003-2311-1814

### Data availability

All of the data that support the findings of this study are available in the main text or Supplementary Information.
